# Galangin mitigates glucocorticoid-induced osteoporosis by activating autophagy of BMSCs via triggering the PKA/CREB signaling pathway: Galangin mitigates glucocorticoid-induced osteoporosis

**DOI:** 10.3724/abbs.2023063

**Published:** 2023-06-26

**Authors:** Chenying Zeng, Shan Wang, Huimin Gu, Fenglei Chen, Ziming Wang, Jinteng Li, Zhongyu Xie, Pei Feng, Huiyong Shen, Yanfeng Wu

**Affiliations:** 1 Center for Biotherapy Eighth Affiliated Hospital of Sun Yat-sen University Shenzhen 518033 China; 2 Department of Orthopedics Eighth Affiliated Hospital of Sun Yat-sen University Shenzhen 518033 China; 3 Department of Orthopedics Sun Yat-sen Memorial Hospital of Sun Yat-sen University Guangzhou 510120 China

**Keywords:** galangin, glucocorticoid-induced osteoporosis, dexamethasone, BMSC, autophagy

## Abstract

Glucocorticoid-induced osteoporosis (GIOP), one of the most common and serious adverse effects associated with glucocorticoid administration, manifests as decreased bone formation and increased bone resorption, eventually culminating in bone loss. Galangin (GAL) is a flavonoid extracted from the medicinal herbal galangal that possesses a variety of pharmacological activities and can inhibit osteoclastogenesis. However, the effects of GAL on GIOP remain unclear. Our study aims to explore the effects of GAL on GIOP in mice and the underlying mechanism. Our results show that GAL markedly mitigates the severity of dexamethasone (Dex)-induced osteoporosis in mice and potentiates osteogenic differentiation in mouse bone marrow-derived mesenchymal stem cells (BMSCs). Furthermore, GAL also significantly counteracts Dex-mediated suppression of osteogenic differentiation and autophagy in human BMSCs. GAL augments PKA/CREB-mediated autophagic flux in BMSCs and the bones of osteoporotic mice. GAL-mediated osteogenic differentiation in Dex-treated BMSCs is significantly decreased by the PKA inhibitor H89 and autophagy inhibitor 3-methyladenine. Collectively, our data indicate that GAL can ameliorate GIOP, partly by augmenting the mineralization of BMSCs by potentiating PKA/CREB-mediated autophagic flux, highlighting its potential therapeutic use in treating glucocorticoid-related osteoporosis.

## Introduction

Glucocorticoids can regulate the synthesis and metabolism of fat and protein and are commonly used in the clinic to inhibit the immune response and inflammation and treat shock [ 1, 2] . However, long-term administration of glucocorticoids can cause osteoporosis [ 3, 4] , particularly when high doses of dexamethasone (Dex), one of the commonly used glucocorticoids, are taken for a long period of time [5]. Currently, drugs that inhibit bone resorption and promote bone formation are mainly used to treat GIOP in the clinic, but these treatments have many side effects. Long-term use of bone resorption inhibitors, such as bisphosphonates, can increase the risk of osteomyelitis and affect bone structure [6]. In addition, the use of bone-promoting drugs, including parathyroid hormone and fluoride, can increase the risk of tumorigenesis, and their safety is still controversial [ 7, 8] . Therefore, there is an urgent need for more effective regimens for GIOP prevention and treatment. Effective prophylactic and therapeutic treatment of GIOP has become an urgent demand in clinical practice.

Bone marrow-derived mesenchymal stem cells (BMSCs) are mesenchymal-derived multipotent stem cells that can promote the structural and functional repair of damaged and aging organs and differentiate into cartilage, bone and adipose tissue under certain conditions [ 9, 10] . Among these, osteogenic differentiation in BMSCs has received particular attention because of its crucial roles in bone formation [11] Although previous studies showed that glucocorticoids could induce osteoporosis by promoting the function of osteoclasts [12], recent studies revealed that glucocorticoids could influence the differentiation and mineralization of osteoblasts, thereby generating reduced bone formation [ 13, 14] . Notably, compared to their enhancement of osteoclast function, glucocorticoids have been shown to predominantly exert their inhibitory effects on the differentiation of BMSCs [15]. Therefore, it is of great importance to search for novel pharmacological agents that can regulate the osteogenic differentiation of BMSCs, which may provide new ways to prevent and treat GIOP.

Autophagy is a self-digesting process in which protein aggregates, damaged organelles, and lipid vesicles are isolated and wrapped in autophagosomes, which then fuse with lysosomes to degrade their contents [16]. Previous studies have shown that autophagy maintains bone homeostasis, and autophagy disorders can cause bone loss and osteoporosis [ 2, 17] . Recent studies have shown that glucocorticoids can reduce the number of osteoblasts and increase bone fragility by inhibiting autophagy [18]. The activation of autophagy in BMSCs could enhance their osteogenic differentiation and promote osteogenesis [19]. Therefore, promoting osteogenic differentiation by regulating autophagy in BMSCs may be a potential strategy for treating GIOP.

In recent years, traditional Chinese medicine (TCM) has been widely used in the treatment and prevention of osteoporosis [20]. A large number of studies have revealed that active components isolated from TCM medicinal herbs exert potential therapeutic effects on GIOP [ 21, 22] . Galangin (GAL, 3,5,7-trihydroxyflavone), a well-known flavonoid extracted from the roots of the medicinal herb galangal that is used in TCM, possesses multifarious pharmacological properties, including anti-inflammatory, antibacterial, antiviral and antitumour activities [ 23‒ 25] . It has been reported that GAL can inhibit osteosarcoma by promoting osteogenic differentiation via the transforming growth factor-β1 pathway [26]. GAL has also been shown to suppress osteoclastogenesis by inhibiting the activation of MAPK and NF-κB signals [ 27, 28] , suggesting the potential for mitigating osteoporosis. However, the pharmacological effects and potential mechanisms of GAL in the treatment of GIOP remain unclear.

In the present study, our goal was to explore the pharmacological effect of GAL on GIOP and clarify the possible mechanism underlying this effect. Our results showed that GAL significantly mitigated the severity of Dex-induced osteoporosis *in vitro* and *in vivo*. Importantly, GAL enhanced the differentiation and mineralization of Dex-treated BMSCs by potentiating PKA/CREB-induced autophagic flux, indicating that GAL may be a potential drug for the treatment of GIOP.

## Materials and Methods

### Drugs and reagents

GAL (S31448; ≥98% purity) was the product of Shanghai Yuanye Biotechnology Company (Shanghai, China), dissolved in DMSO to prepare a 100 mM stock solution and diluted to different working concentrations with cell culture medium. Trypsin (BL501A) was obtained from Biosharp (Guangzhou, China). Dexamethasone (Dex; D4902) and 3-methyladenine (3-MA; M9281) were purchased from Sigma (St Louis, USA). Alizarin Red S (ARS; G8550) was the product of Solarbio (Shanghai, China). An alkaline phosphatase assay kit (ALP; A059-2-2-96T) was purchased from Jiancheng Biotech (Nanjing, China). SDS-PAGE preparation kits (P0012A), 5× SDS-PAGE protein loading buffer (P0286), BCIP/NBT ALP colour development kit (C3206), H89 (S1643), antibodies against p-(Ser/Thr), PKA substrate (AF9621), and anti-GAPDH antibody (AF5009) were the products of Beyotime Biotechnology (Shanghai, China). Antibodies against Runx2 (8486) and p62/SQSTM1 (39749S) were the products of CST (Beverly, USA). Antibodies against osteopontin (OPN; ab69498), osteocalcin (OCN; ab93876), LC3B (ab192890), CREB (ab32515), and p-CREB (ab254107) were purchased from Abcam (Cambridge, UK). Antibody against beclin-1 (sc-48381) was the product of Santa Cruz (Santa Cruz Biotech, USA). HRP-conjugated goat anti-rabbit IgG (G1213) and HRP-conjugated horse anti-mouse IgG (G1214) were obtained from Servicebio (Wuhan, China). PVDF membranes (IPVH00010) were the products of Millipore (Billerica, USA).

### Animals

Three-month-old male C57BL/6 mice were obtained from the Laboratory Animal Centre of Sun Yat-sen University (Guangzhou, China), housed in rearing cages and provided with water and food independently. All animal experiments were carried out with the approval by the Animal Ethics Committee of Sun Yat-sen University (SYSU-IACUC-2022-001067).

### Isolation and culture of human BMSCs

In this study, we selected 15 healthy volunteers and asked them to sign informed consent forms for the extraction of bone marrow. The isolation and culture of BMSCs were performed as described previously [ 29, 30] . Briefly, the bone marrow of volunteers was extracted under aseptic conditions, and then the BMSCs were isolated and cultured in DMEM medium (Gibco, Carlsbad, USA) containing 10% FBS for 48 h. Subsequently, to remove the unattached cells, the medium was replaced every three days. When the cell density reached 90%, BMSCs were subcultured and digested with 0.25% trypsin, and then BMSCs were evenly resuspended and expanded to several new flasks for further culture. Cells were grown to passage 3 for the subsequent studies. This study complied with the ethical standards of the Helsinki Declaration and was approved by the Clinical Research Ethics Committee of the Eighth Affiliated Hospital of Sun Yat-sen University (ZB-KYIRB-2022-021-02).

### Cell viability assay

Human BMSCs were digested, resuspended, inoculated in 96-well plates (5000 cells/well), and cultured overnight. Then, the BMSCs were treated with 5, 10, 20, 40, and 80 μM GAL for 24 h and 72 h. After that, CCK-8 reagent was added to BMSCs and incubated for 1 h. Then cell viability was determined by measuring the absorbance at 450 nm.

### Osteogenic differentiation and characterization

Cells were cultivated in osteogenic differentiation medium (DMEM containing 10% FBS, 100 U/mL penicillin, 50 μM ascorbic acid, 100 μg/mL streptomycin and 10 mM β-glycerol phosphate) with or without 0.1 mM Dex for 7 or 14 days. The osteogenic differentiation medium was changed every three days. The level of osteogenic differentiation was analyzed by alkaline phosphatase (ALP) activity analysis and Alizarin Red S (ARS) staining.

ALP analysis was conducted according to the manufacturer’s instructions. In brief, after 7 days of osteogenic induction, BMSCs were rinsed with sterile PBS and fixed with 4% paraformaldehyde, and moderate BCIP/NBT solution (Beyotime Biotechnology) was used to stain cells. Then, the staining liquid was discarded, and the cells were rinsed with PBS three times to stop the reaction. Finally, the ALP activity of osteoblast differentiation was analysed under a microscope. In addition, the ALP activity kit (Jiancheng Biotech) was also used to analyze the ALP activity of the cells. In short, the cells were centrifuged after lysis, and then the cell supernatant was collected and incubated with the reaction solution at 37°C for 15 min. Absorbance was detected at 405 nm after the termination fluid was added.

ARS staining was conducted to analyze mineralization after 14 days of osteogenic culture as described previously [31]. In brief, the cells were rinsed with sterile PBS, fixed with 4% paraformaldehyde, and stained with 1% ARS (Solarbio) at room temperature for 20 min. After that, the cells were photographed and analyzed under a microscope. To measure spectrophotometric absorbance, 10% cetylpyridinium chloride monohydrate was used to decolorize the cells. Finally, the decolorized suspension was transferred to a new 96-well plate, and then the absorbance was measured at 562 nm with a spectrophotometer.

### Treatment of human BMSCs

GAL was dissolved in DMSO as a 100 mM stock solution and then diluted to different concentrations (0, 5, 10, and 20 μM) with osteogenic medium at the final working concentrations. To evaluate the influence of GAL on the osteoblast differentiation of Dex-treated BMSCs, BMSCs were resuspended, seeded in culture plates with DMEM containing 10% FBS, and cultured for 12 h. Then, the culture medium was removed, and the BMSCs were cultured in osteogenic medium supplemented with Dex (1 μM) and gradient concentrations of GAL. The medium was changed every other day.

Based on the experimental settings, the PKA signal inhibitor H89 (Beyotime Biotechnology) was used in the culture system at a concentration of 10 μM. To evaluate the influence of GAL-mediated autophagy induction in Dex-treated BMSCs, the Dex-treated cells were cultured with 3-MA (specific inhibitor of autophagy) at a concentration of 10 mM.

### Autophagosome formation analysis

First, cells were cultured in confocal dishes with DMEM containing 10% FBS overnight. After that, the cells were cultured with DAP Green Working Solution (Dojindo, Tokyo, Japan) for 30 min. Then, the culture supernatant was removed, and the cells were cultured with gradient concentrations of GAL for 24 h. Finally, intracellular autophagosome formation in BMSCs was analyzed under a confocal laser scanning microscope (Leica, Wetzlar, Germany).

### Autophagic flux analysis

MRFP-GFP-LC3 adenovirus (OBiO Technology, Shanghai, China) was used to detect autophagic flux. Briefly, cells were cultured in confocal dishes with DMEM containing 10% FBS overnight. After that, the cells were cultured with fluorescent mRFP-GFP-LC3 adenovirus solution for 24 h. Then, the culture supernatant was removed, and the cells were cultured with gradient concentrations of GAL for 24 h. Finally, intracellular autophagic flux in BMSCs was analyzed with a confocal laser scanning microscope. Images were acquired under a 40× oil objective.

### Western blot analysis

Briefly, the cells were rinsed with sterile PBS and then lyzed with RIPA lysis buffer (AR0105; Boster, Wuhan, China) containing protease and phosphatase inhibitors. Then, the protein concentration was analyzed using a BCA protein assay kit (CW0014; CWBIO, Taizhou, China). Then, equivalent amounts of protein were separated by SDS-PAGE and transferred onto PVDF membranes. Then, the membranes were blocked and incubated with the specified primary antibodies at 4°C overnight. Subsequently, the membranes were incubated with HRP-conjugated secondary antibodies. Finally, the levels of protein expression were detected using chemiluminescent reagents (WBKLS0500; Millipore).

### Immunofluorescence microscopy

In brief, BMSCs were cultured on glass coverslips and treated based on the experimental design. Mouse femurs were decalcified in 10% EDTA for 10 days, and the solution was changed every three days. After that, the bones were embedded in paraffin, sectioned (5 mm thickness), and stained for immunofluorescence analysis. After the fixation, permeabilization and blocking processes, the cells and femur bones were incubated with specific primary antibodies (1:300) overnight and then stained with specific secondary antibodies (4413; 1:500; CST) at room temperature. Finally, images were acquired with a confocal imaging system (Carl Zeiss, Oberkochen, Germany).

### GIOP animal model establishment and drug treatment

The murine GIOP model was established as described previously [31]. In brief, mice were randomly assigned into four groups, and then a dose of 2 mg/kg Dex was injected into the hind leg muscle three times a week for eight weeks to establish the GIOP mouse model. The drug GAL was dissolved and diluted by PBS containing 2% Tween-80, and then mice were intragastrically administered with GAL (10 or 40 mg/kg) or vehicle (2% Tween-80 in PBS) once a day for eight weeks, which was based on previous studies [ 28, 32] . The mice were sacrificed after eight weeks, and the serum, BMSCs and femurs were acquired for further analysis.

### Isolation and culture of murine BMSCs

The femurs of GIOP mice were rinsed with sterile PBS to obtain cell suspensions. Then, the cell suspensions were collected and centrifuged. The cell pellets were cultured in specific culture medium (05513-1Kit; Stem Cell Mesen Cult Expansion kit, Vancouver, Canada) containing 100 U/mL penicillin and 100 μg/mL streptomycin. After the cell density reached 90%, the cells were trypsinized and subcultured. BMSCs at passage 3 were used for the subsequent experiments.

### Flow cytometric analysis of BMSCs

BMSCs at passage 3 were used for flow cytometric analysis. Human BMSCs were incubated with primary antibodies against HLA-DR, CD105, CD90, CD73, CD14, CD45, and CD34. Murine BMSCs were incubated with antibodies against Sca-1, CD45, CD44, CD29, and CD34. All antibodies used in flow cytometry were purchased from BD Biosciences (Franklin Lakes, USA). Finally, cells were analyzed with a FACSCalibur flow cytometer (BD Biosciences).

### Micro-CT measurement

The femur of the femora was accessed by micro-CT. The femurs were fixed with 4% paraformaldehyde for 48 h, after which they were scanned and analyzed. The images were acquired with the following parameters: an effective pixel size of 8.82 lm, voltage of 80 kV, exposure time of 1500 ms, and current of 500 lA. The bone volume/total volume (BV/TV), trabecular thickness, number and spacing of the trabecular area were evaluated by the Inveon Research Workplace (Siemens, Munich, Germany) according to introductions developed by the American Society for Bone and Mineral Research.

### Haematoxylin & eosin (H&E) staining and tartrate-resistant acid phosphatase (TRAP) staining

After fixation in 4% polyoxymethylene for 48 h, the femurs were decalcified in 10% EDTA for 10 days. After being embedded in paraffin and sliced into sections, the femurs were stained. TRAP staining and H&E staining were performed using the corresponding kits (BP088; Biossci, Wuhan, China and AR1180; Boster) according to the manufacturer’s specifications, and the number of TRAP-position osteoclasts (OCs) and OCs per bone surface (OC/BS) was determined by ImageJ software (NIH, Bethesda, USA).

### Statistical analysis

Data were presented as the mean±standard deviation (SD) and assessed for statistical significance using GraphPad Prism 8.0. The statistical differences among multiple groups and between two groups were calculated with one-way analysis of variance (ANOVA) followed by Tukey’s post hoc test and unpaired Student’s *t* test, respectively. *P*<0.05 was defined as statistically significant.

## Results

### GAL attenuated Dex-induced osteoporosis in mice

**Figure 1:**
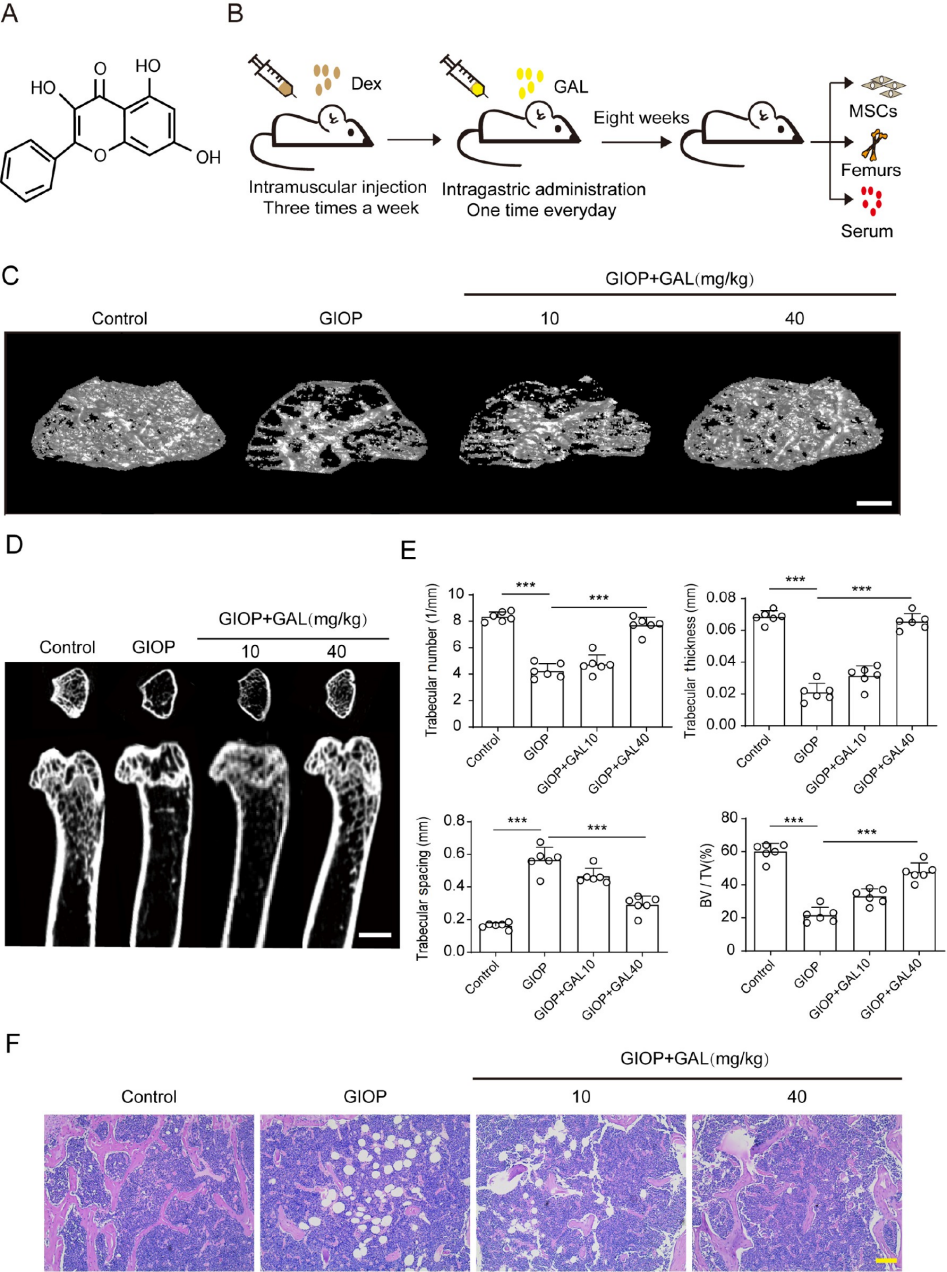
Figure 1 Galangin (GAL) alleviated dexamethasone (Dex)-induced bone damage in mice (A) Two-dimensional structure of GAL. (B) Flowchart of glucocorticoid-induced osteoporosis (GIOP) mouse experiments. Dex was intraperitoneally administered three times a week, and GAL was gavaged once a day. The whole experiment lasted for eight weeks. (C) Representative micro-CT analysis of the three-dimensional reconstruction graph for different groups: control ( n=6), GIOP ( n=6), GIOP +10 mg/kg GAL ( n=6) or GIOP +40 mg/kg GAL ( n=6). Scale bar: 1 mm. (D) Micro-CT analysis of femurs from mice of different groups: Control ( n=6), GIOP ( n=6), GIOP +10 mg/kg GAL ( n=6) or GIOP +40 mg/kg GAL ( n=6). Scale bar: 1 mm. (E) BV/TV, trabecular thickness, trabecular number, and trabecular spacing analysis of femurs from mice of different groups. (F) H&E staining of femurs from mice of different groups: Control ( n=6), GIOP ( n=6), GIOP +10 mg/kg GAL ( n=6) or GIOP +40 mg/kg GAL ( n=6). Scale bar: 500 μm. Data are shown as the mean±SD. *** P<0.001. GIOP: glucocorticoid-induced osteoporosis.

We initially investigated whether GAL had a protective effect on GIOP mice induced by Dex. The molecular structure of GAL is presented in Figure 1A, and the administration process of mice is shown in Figure 1B. We performed H&E staining and micro-CT to investigate the effects of GAL on Dex-treated mice. The results showed that mice treated with Dex exhibited less mineralization and bone trabeculae and more adipocytes in the femurs than those in the control group ( Figure 1C,D). However, GAL (10 to 40 mg/kg) observably increased bone mineralization and bone trabeculae and reduced adipocytes in Dex-treated mice, as shown by H&E staining ( Figure 1F). Furthermore, the micro-CT results showed that Dex significantly increased trabecular spacing and decreased trabecular number, trabecular thickness, bone mineralization and BV/TV, and GAL treatment significantly reversed these changes in Dex-treated mice ( Figure 1E). Taken together, these results indicated that GAL could alleviate Dex-induced osteoporosis in mice.

### GAL augmented osteogenic differentiation in BMSCs in Dex-treated mice

**Figure 2:**
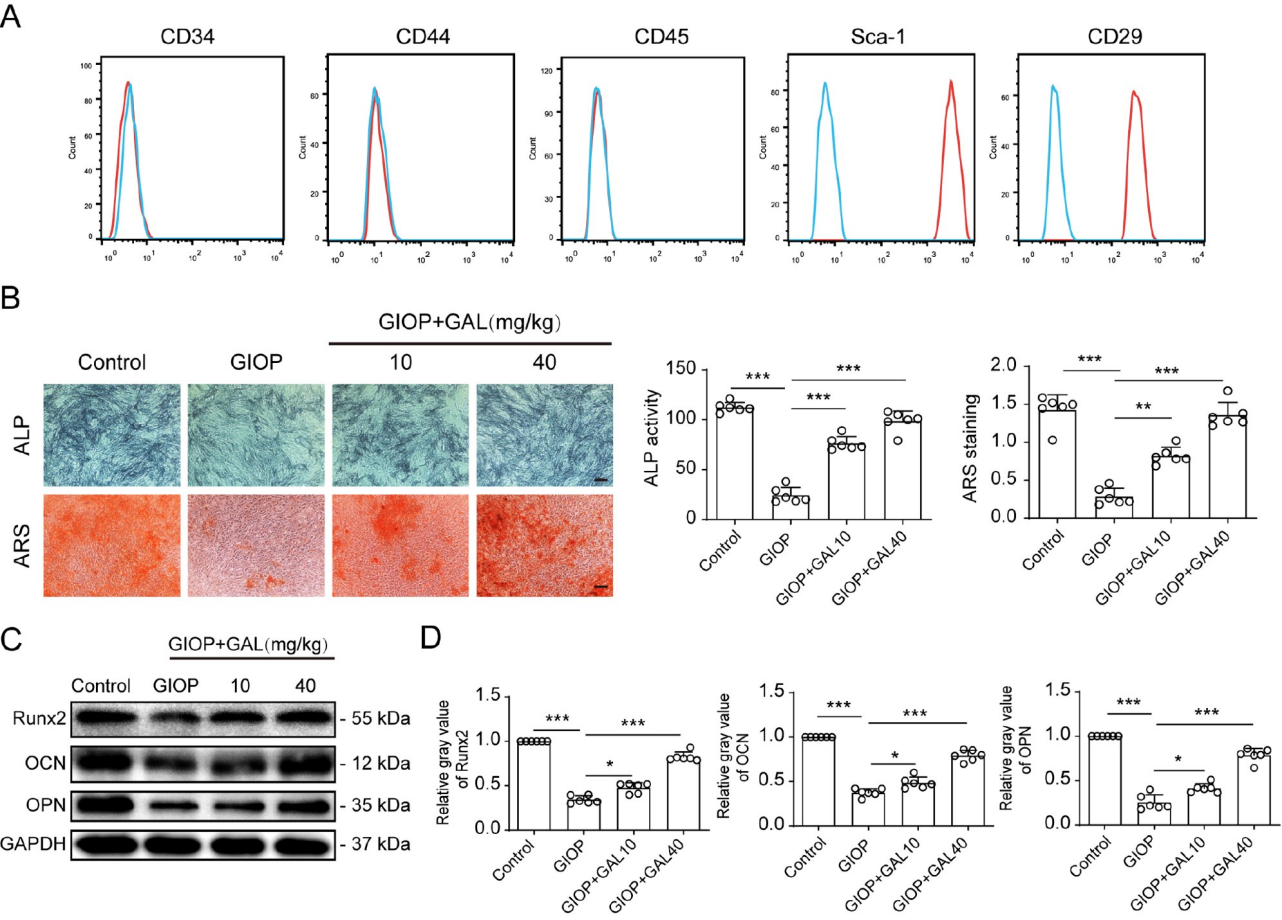
Figure 2 GAL promoted osteogenic differentiation of bone marrow-derived mesenchymal stem cells (BMSCs) in Dex-treated mice (A) Murine BMSCs were negative for CD44, CD34, and CD45 expressions and positive for CD29 and Sca-1 expressions. (B) BMSCs of different mouse groups were treated with osteogenic medium for 7 and 14 days, and then alkaline phosphatase (ALP) activity assay and Alizarin red S (ARS) staining for calcium deposition were performed as described in “Materials and Methods”. Scale bar: 100 μm. (C) Western blot analysis of BMSCs after osteogenic differentiation indicated that BMSCs of GAL-treated mice increased the expression levels of the osteogenic protein markers Runx2, osteocalcin (OCN), and osteopontin (OPN), and the relative gray values are shown in the histograms (D). Data are shown as the mean±SD ( n=6). * P<0.05, ** P<0.01, *** P<0.001. GIOP: glucocorticoid-induced osteoporosis.

It is well known that an imbalance between osteoblasts and osteoclasts in bone metabolism leads to the progression of osteoporosis. GAL has been reported to suppress LPS-induced bone resorption by inhibiting osteoclastogenesis [ 27, 28] . We examined osteoclast function in mice by TRAP staining, and the staining was increased in Dex-treated mice in comparison with that in normal mice, and the staining was decreased in Dex+GAL-treated mice compared with that in Dex-treated mice ( Supplementary Figure S1), which was consistent with previous literature [27]. Recent studies have found that glucocorticoids are closely related to the differentiation and mineralization of BMSCs, which can reduce the mineralization of BMSCs and promote adipogenic differentiation of BMSCs, eventually leading to bone loss [15]. We then examined whether GAL could affect the differentiation and mineralization of BMSCs in Dex-treated mice. BMSCs were harvested from the femurs of treated mice and the BMSC phenotypes were identified by flow cytometry, and the results indicated that CD34, CD44 and CD45 were negative in the cells, and CD29 and Sca-1 were positive, which indicated the correct phenotype of mouse BMSCs ( Figure 2A). ALP activity assays and ARS staining were used to evaluate the differentiation and mineralization of BMSCs. The results showed weaker ALP activity and lower mineralization in BMSCs from Dex-treated mice than in those from control mice, and GAL treatment reversed the negative effects of Dex ( Figure 2B). In addition, GAL treatment increased the expressions of Runx2, OCN, and OPN, which are marker proteins of osteogenic differentiation ( Figure 2C,D). These results revealed that GAL could reverse the decreased osteogenic mineralization of BMSCs in GIOP mice, suggesting that GAL alleviated GIOP by influencing the level of osteogenic differentiation in BMSCs.

### GAL counteracted Dex-induced inhibition of osteogenic differentiation in human BMSCs

As GAL could augment osteogenic mineralization of BMSCs in GIOP mice, we next probed the potential mechanism by which GAL alleviates GIOP by using Dex-treated human BMSCs as the cell model. The phenotype and differentiation potential of human BMSCs were determined. The results revealed that HLA-DR, CD45, CD34 and CD14 were negative, while CD105, CD73, and CD90 were positive in the cells ( Supplementary Figure S2A), which conformed to the criteria of the International Society for Stem Cell Research. At the same time, the isolated BMSCs were able to differentiate into chondroblasts, osteoblasts, and adipocytes under appropriate conditions ( Supplementary Figure S2B). CCK-8 analysis indicated that GAL had no cytotoxicity or had only weak cytotoxicity on the cells at the tested concentrations (5, 10, and 20 μM) after 24 h and 72 h of incubation ( Supplementary Figure S2C). Thus, these concentrations (5, 10, and 20 μM) were used for most of the subsequent experiments.

**Figure 3:**
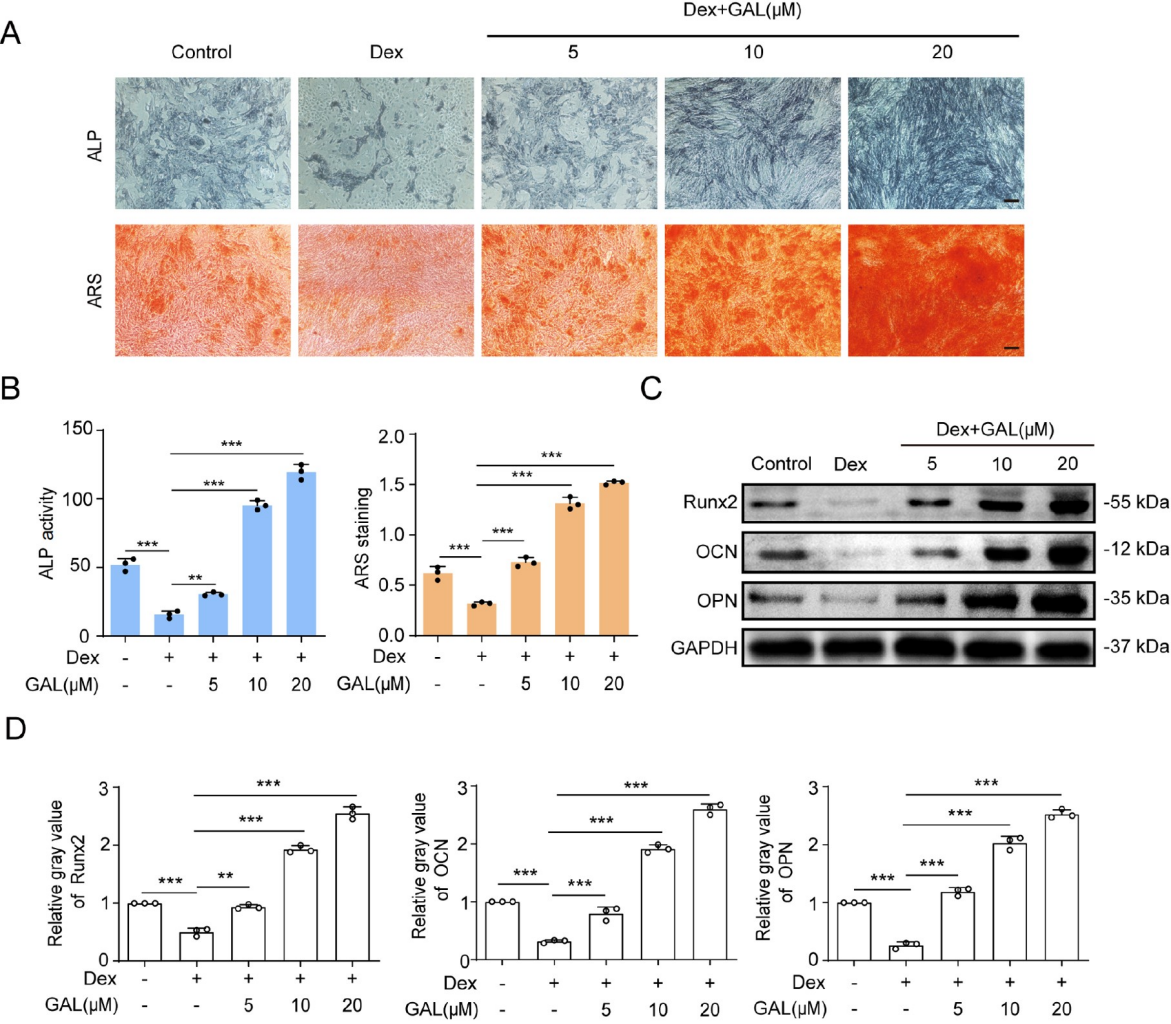
Figure 3 GAL reversed the suppressive effect of Dex on the osteogenic differentiation of human BMSCs (A,B) BMSCs were treated with graded doses of GAL in combination with Dex (1 μM) in osteogenic medium for 7 days and 14 days and then subject to ALP activity assay and ARS staining. Scale bar: 100 μm. (C) The protein expression levels of Runx2, OCN, OPN and GAPDH were determined by western blot analysis, and the gray values relative to GAPDH are shown in the histograms (D). Data are shown as the mean±SD ( n=3). ** P<0.01, *** P<0.001.

First, we investigated whether GAL could affect the differentiation and mineralization of human BMSCs. As expected, GAL administration markedly enhanced the osteogenic ability of human BMSCs in a dose-dependent manner (Supplementary Figure S3). In the ALP activity assay and ARS staining, human BMSCs treated with Dex exhibited an obvious decrease in ALP activity and mineralization levels. Interestingly, GAL (from 5 to 20 μM) was able to dramatically increase ALP activity and mineralization, indicating enhanced osteogenic differentiation, which is consistent with the results of BMSCs in GIOP mice ( Figure 3A,B). Moreover, western blot analysis showed that GAL treatment alleviated Dex-induced downregulation of Runx2, OPN, and OCN expressions in Dex-treated human BMSCs ( Figure 3C,D). These results showed that GAL could significantly facilitate the differentiation and mineralization of GIOP BMSCs.

### GAL strengthened autophagic flux in Dex-treated human BMSCs

**Figure 4:**
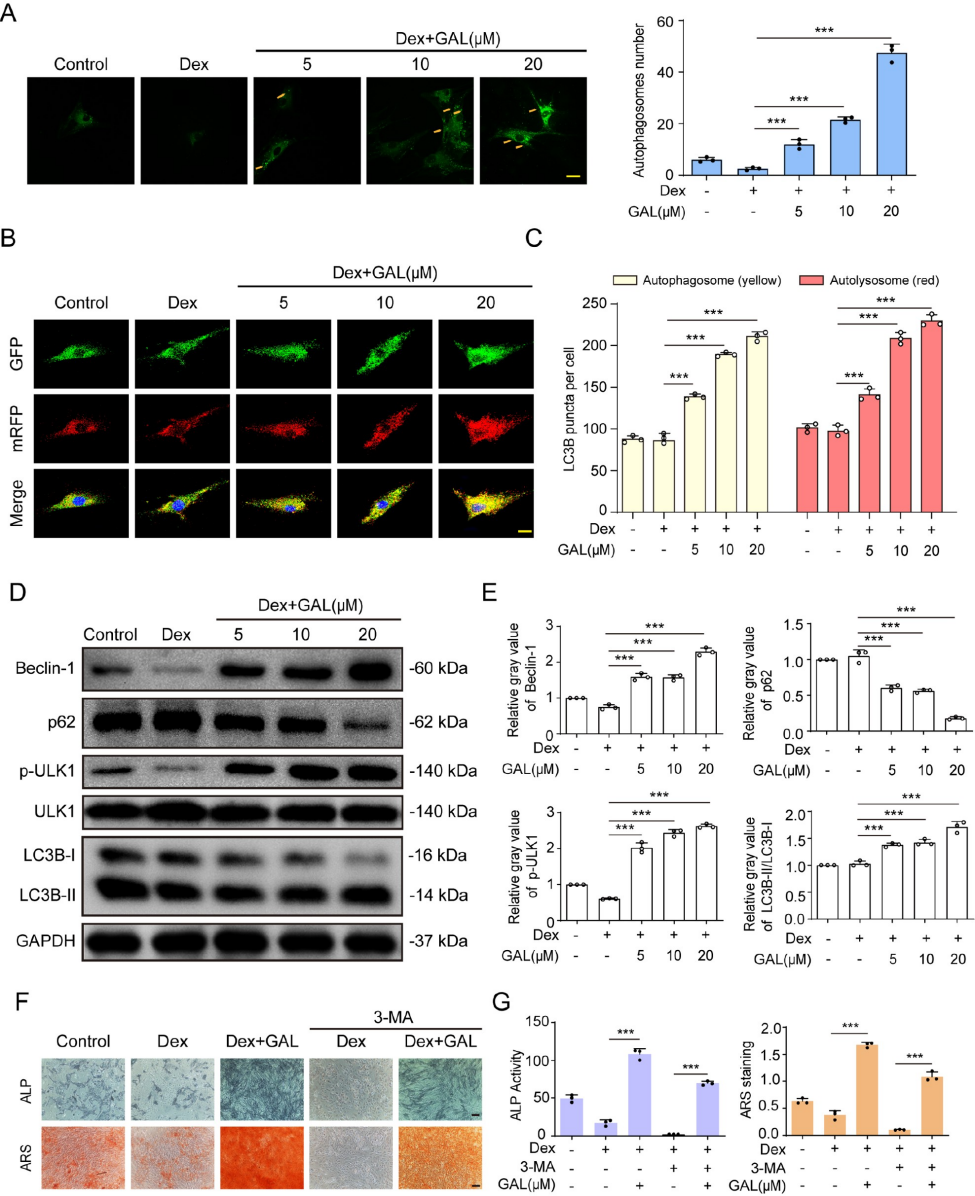
Figure 4 GAL increased autophagic flux in Dex-treated human BMSCs (A) Immunofluorescence microscopy showing autophagosome formation in BMSCs treated with Dex (1 μM) in combination with GAL (0,5,10, and 20 μM) for 24 h. Scale bar: 20 μm. (B,C) GFP-mCherry-LC3B double label overexpression lentivirus immunofluorescence staining of LC3B expression in BMSCs after 24 h of Dex (1 μM) and GAL (0,5,10, and 20 μM) cotreatment. Scale bar: 20 μm. (D) The expressions of beclin-1, LC3B, p-ULK-1, and p62/SQSTM1 were measured by western blot analysis, and the relative gray values are shown in the histograms (E). (F,G) Cells were pretreated with 3-MA (10 mM) for 24 h and then treated with Dex (1 μM) and GAL (20 μM) in osteogenic medium for 7 days and 14 days, followed by analysis of ALP activity and ARS staining. Data are shown as the mean±SD (n=3). *** P<0.001.

We next sought to examine the possible mechanism of GAL in Dex-treated BMSCs. Previous studies have revealed that glucocorticoids can inhibit autophagy in BMSCs, which is related to the pathogenesis of GIOP [ 33, 34] . In addition, GAL has been shown to activate autophagy under specific induction conditions [35]. Based on these previous findings, we examined whether GAL could enhance autophagy flux levels in Dex-treated human BMSCs. The results showed that GAL promoted autophagosome formation at the concentrations of 5, 10, and 20 μM in a dose-dependent manner in Dex-treated human BMSCs ( Figure 4A). Furthermore, we used GFP-mCherry-LC3B double label overexpression lentivirus to further examine whether GAL could enhance autophagy flux levels in Dex-treated human BMSCs. The results showed that GAL treatment promoted the formation of autophagosomes (yellow puncta in merged image) and autolysosomes (red puncta in merged image) at the concentrations of 5, 10, and 20 μM in a dose-dependent manner in Dex-treated human BMSCs ( Figure 4B,C). Furthermore, we examined other autophagy-related signalling pathway molecules. Consistent with our expectations, GAL significantly reversed the inhibitory effect of Dex on autophagy-related LC3B, ULK-1, beclin-1 and p62/SQSTM1 expressions in Dex-treated BMSCs ( Figure 4D,E). We next used the autophagy inhibitor 3-MA to explore whether autophagy is effective in osteoblast differentiation in Dex-treated human BMSCs. BMSCs were treated with 20 μM GAL followed by Dex treatment in the presence or absence of 3-MA. Interestingly, 3-MA reversed GAL-mediated promotion of ALP activity and osteogenic mineralization in Dex-treated BMSCs ( Figure 4F,G). Taken together, these results indicated that GAL-induced autophagy signalling can be a possible mechanism for the protective effect of GAL to mitigate osteogenic differentiation in Dex-treated human BMSCs.

### PKA/CREB-mediated signaling pathway is involved in the augmenting effect of GAL-induced autophagy in Dex-treated human BMSCs

**Figure 5:**
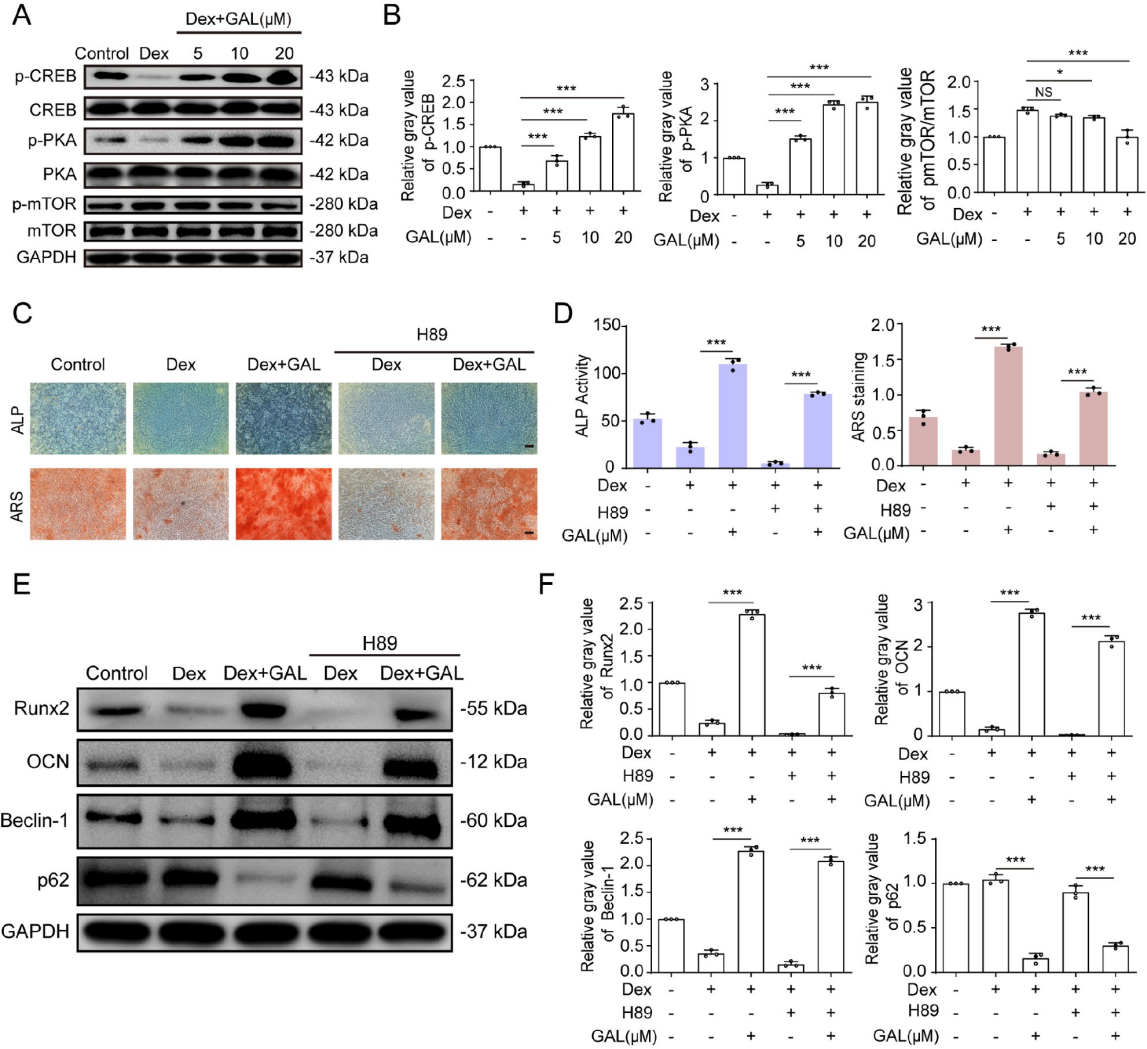
Figure 5 GAL activated the PKA/CREB-mediated autophagy pathway in Dex-treated human BMSCs (A) Western blot analysis was used to detect the protein expressions of p-CREB, p-PKA, and p-mTOR, and the relative gray values are shown in the histograms (B). (C,D) Cells were pretreated with H89 (10 μM) for 24 h and then treated with Dex (1 μM) and GAL (20 μM) in osteogenic medium for 7 days and 14 days. The cells were analyzed for ALP activity and ARS staining. Scale bar: 100 μm. (E) Western blot analysis was used to detect the expressions of Runx2, OCN, beclin-1, and p62/SQSTM1, and the relative gray values are shown in the histograms (F). Data are shown as the mean±SD ( n=3). * P<0.05, *** P<0.001. NS: not significant.

Next, we investigated the specific molecular signalling pathway by which GAL promotes autophagy in Dex-treated human BMSCs. Western blot analysis was used to detect the classical autophagy signalling pathway, such as PKA/CREB, AKT/mTOR, PI3K/AKT, and Wnt/β-catenin. Our results showed that GAL observably upregulated the levels of p-PKA/PKA and p-CREB/CREB and downregulated the level of p-mTOR/mTOR in the Dex+GAL groups compared to those in the Dex group ( Figure 5A,B). Furthermore, H89 (a specific inhibitor of PKA) attenuated the enhanced mineralization decreased by GAL in Dex-treated BMSCs ( Figure 5C,D). In addition, treatment with H89 not only decreased the levels of the osteogenic proteins Runx2 and OCN but also decreased the levels of the autophagy-related proteins beclin-1 and p62/SQSTM1 ( Figure 5E,F). Taken together, these results indicated that the PKA/CREB-mediated signalling pathway is required for GAL to enhance autophagy signalling in Dex-treated human BMSCs.

### GAL administration activated PKA/CREB-mediated autophagy in GIOP mice

**Figure 6:**
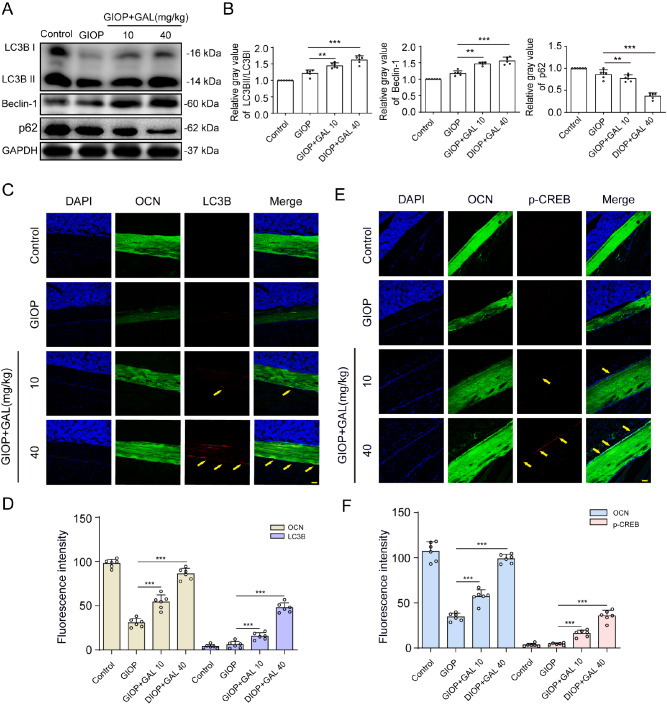
Figure 6 GAL administration activated PKA/CREB-mediated autophagy in GIOP mice (A,B) BMSCs from mice of different groups were treated with osteogenic medium, and then western blot analysis was used to detect the protein expressions of LC3B, beclin-1, and p62/SQSTM1. (C,D) Representative immunofluorescence images showing that GAL at 40 mg/kg significantly upregulated LC3B expression level in the femurs of GIOP mice (yellow arrows). Scale bar: 50 μm. (E,F) Immunofluorescence staining demonstrated that the expression of p-CREB was enhanced in GIOP+GAL-treated femurs compared with those in the control and GIOP femurs (yellow arrows). Scale bar: 50 μm. Data are shown as the mean±SD ( n=6). ** P<0.01, *** P<0.001.

Because PKA/CREB-mediated autophagy is critical for GAL-mediated remission of bone mineralization in Dex-treated human BMSCs, we further examined whether GAL could activate autophagy in GIOP mice. Interestingly, treatment with Dex obviously decreased the expressions of beclin-1 and LC3B, but GAL reversed the inhibitory effect of Dex on BMSCs in GIOP mice ( Figure 6A,B). Moreover, immunofluorescence analysis showed that LC3B and p-CREB levels were increased in Dex+GAL-treated mice compared to those in Dex-treated mice ( Figure 6C‒F), indicating enhanced autophagy in GAL-treated GIOP mice. These results indicated that GAL could enhance PKA/CREB-mediated autophagy signalling in GIOP mice, which was associated with the alleviation of the severity of Dex-induced osteoporosis.

## Discussion

GIOP is the secondary cause of osteoporosis, which leads to bone loss and bone fracture [36]. In recent years, a large amount of evidence has shown that GIOP is caused by glucocorticoid-mediated inhibition of new bone formation by attenuating the proliferation, differentiation, and mineralization of osteoblasts [ 14, 37] . BMSCs have the ability to undergo multidirectional differentiation and can be differentiated into osteoblasts under certain conditions. Previous studies have revealed that GIOP can affect the differentiation and function of BMSCs, leading to a decrease in bone formation rate [38]. Recently, it has been shown that glucocorticoids play a key role in the formation and development of GIOP by inhibiting the differentiation and function of BMSCs [39]. In addition, the occurrence of GIOP is linked to decreased autophagy levels in osteoblasts, resulting in reduced osteogenic differentiation [34]. These recent findings suggest that glucocorticoids may induce osteoporosis by interfering with osteogenic differentiation.

Considering that glucocorticoid administration is connected to the dysregulation of new bone formation and decreased osteogenic differentiation in BMSCs, which represents a new pathological mechanism for GIOP [ 13, 15] , we further examined the influence of GAL on differentiation and mineralization *in vivo* and *in vitro* by measuring the expressions of Runx2, OPN and OCN. The results demonstrated that GAL markedly promoted osteogenic differentiation in BMSCs in Dex-treated mice. In addition, human BMSCs were cultured with Dex (1 μM) as the *in vitro* GIOP cellular model. The results showed that Dex could significantly weaken the osteogenic differentiation of human BMSCs, which is consistent with previously published results [40]. Interestingly, GAL also significantly promoted osteogenic differentiation in Dex-treated human BMSCs. To the best of our knowledge, we are the first to uncover the pharmacological effect of GAL on the osteogenic differentiation and mineralization of GIOP and Dex-treated BMSCs, which warrants further clinical investigation.

Recent studies have revealed that GIOP is closely connected to autophagy and that autophagic flux can influence the occurrence and development of GIOP through osteogenic signalling pathways, thereby regulating the balance between bone resorption and bone formation [ 41, 42] . Interestingly, it has been shown that small molecule flavonoids, including GAL, can induce autophagy to various degrees in many cellular models [ 43, 44] . Therefore, we hypothesized that GAL may enhance autophagic flux to potentiate BMSC differentiation towards osteoblasts. In support of this hypothesis, our results showed that GAL promoted autophagic flux during osteogenic differentiation in BMSCs, as evidenced by increased formation of autophagosomes and decreased levels of p62/SQSTM1. Moreover, the immunofluorescence results indicated that LC3B was elevated in the bones of GAL-treated GIOP mice compared with that in GIOP mice. These findings suggest that increased autophagic flux is the mechanism by which GAL mitigates the severity of GIOP in mice and Dex-treated human BMSCs.

Based on these results, we examined the signaling pathways related to autophagy and osteogenesis. Our results revealed that GAL significantly enhanced PKA/CREB signalling in BMSCs, which is likely responsible for autophagy induction and the augmentation of osteogenic differentiation. PKA/CREB signalling has been shown to not only play a key role in osteogenic differentiation but also modulate autophagy induction [ 45, 46] . To confirm the pivotal role of PKA/CREB-mediated signalling in the autophagy-inducing effect of GAL, were used both the autophagy inhibitor 3-MA which abrogated GAL-mediated augmentation of osteogenic differentiation and the PKA inhibitor H89 which attenuated GAL-mediated promotion of osteogenesis in Dex-treated human BMSCs, accompanied by decreased induction of autophagy. In addition, oral administration of GAL increased autophagy induction in BMSCs and the bones of GIOP mice. Our data indicate that GAL can counteract the inhibitory effect of Dex on osteogenic differentiation by modulating PKA/CREB-mediated autophagy.

**Figure 7:**
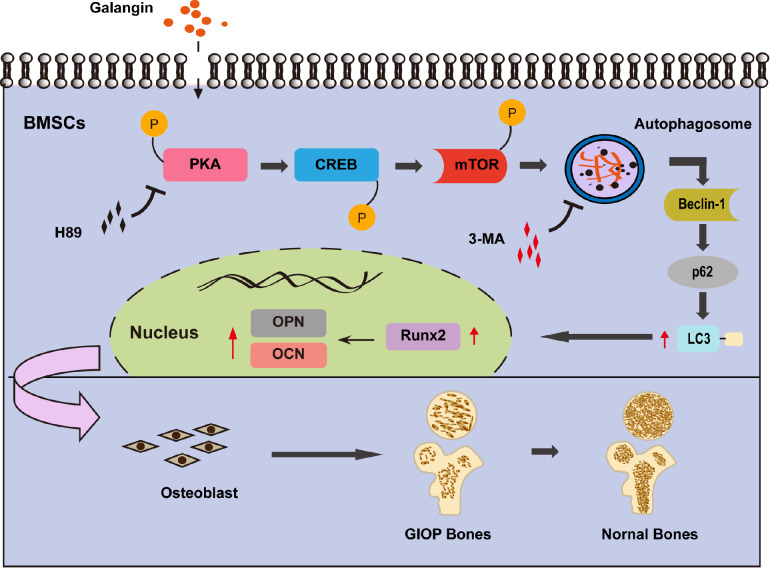
Figure 7 Proposed model for the alleviative effects of GAL against GIOP GAL upregulates the expressions of Runx2, OCN, and OPN proteins in BMSCs by activating PKA/CREB-mediated autophagy, promoting osteogenic differentiation of BMSCs, and ultimately alleviating GIOP.

In conclusion, we showed that the natural flavonoid GAL could effectively mitigate the development and progression of GIOP, at least partially, by augmenting osteogenic differentiation in BMSCs via promoting PKA/CREB-mediated autophagy induction ( Figure 7). Our study provides an experimental basis for the clinical application of TCM for treating GIOP and lays a research foundation for the development of new drugs related to GAL and autophagy for the treatment of GIOP. Although human BMSCs were used in this study and thus indicated translational significance, further preclinical and clinical investigations are still warranted to validate the pro-osteogenesis activities of GAL in humans.

## Supporting information

22698supplementary_data-z

## Supplementary Data

Supplementary data is available at *Acta Biochimica et Biophysica Sinica* online.
