# Supplementary material for: Galangin mitigates glucocorticoid-induced osteoporosis by activating autophagy of BMSCs via triggering the PKA/CREB signaling pathway: Galangin mitigates glucocorticoid-induced osteoporosis
Source: Acta Biochim Biophys Sin (Shanghai). 2023 Jun 26;55(8):1275–87. doi: 10.3724/abbs.2023063 (PMC10448057; doi:10.3724/abbs.2023063)
Supplement: 22698supplementary_data-z [file 22698supplementary_data-z.pdf]

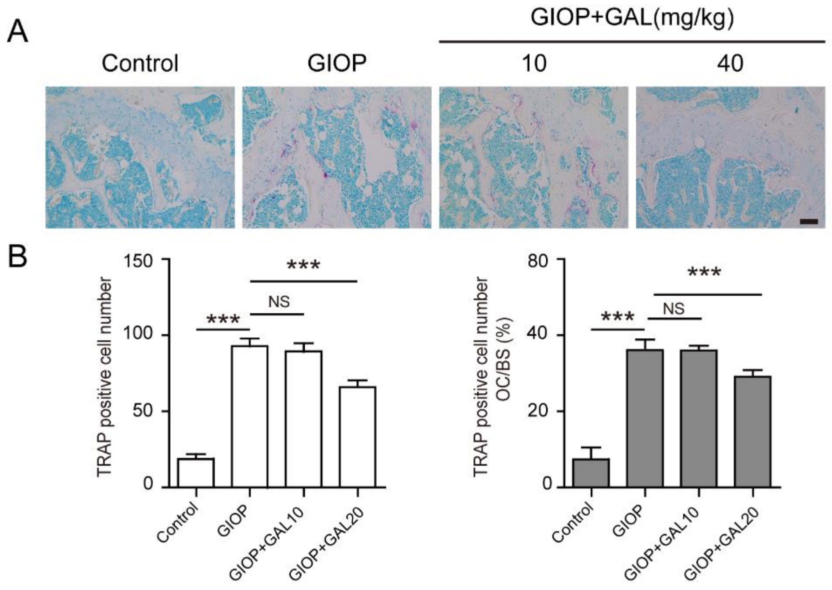

Supplementary Figure S1. TRAP analysis of the effect of GAL on GIOP mice (A) Representative TRAP staining of distal femoral pictures, Scale bar: 100 μm. (B) The number of TRAP-positive cells and OC/BS% were detected by ImageJ, and GAL had a minimal effect on osteoclast differentiation in GIOP mice. Data are shown as the mean ± SD (*n* = 3). ****P* < 0.001. NS: not significant.

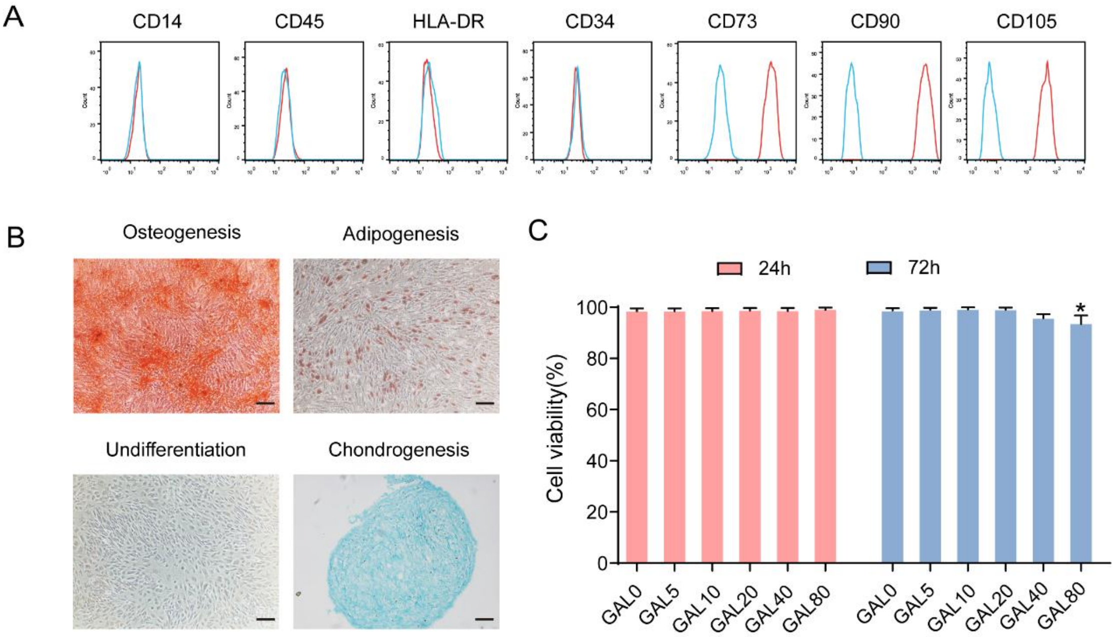

Supplementary Figure S2. Phenotypic identification of human BMSCs and the effect of GAL on the viability of human BMSCs (A) Human BMSCs were negative for CD34, CD14, CD45, and HLA-DR expressions and positive for CD105, CD73 and CD90 expressions. (B) Human BMSCs were subject to chondrogenic differentiation, osteogenic differentiation, and adipogenic differentiation. Scale bar: 250 μm. (C) CCK8 assay was performed to determine the cytotoxicity of GAL on human BMSCs after

treatment for 24 h and 72 h. Data are shown as the mean ± SD (n = 3). $^{*}P < 0.05$.

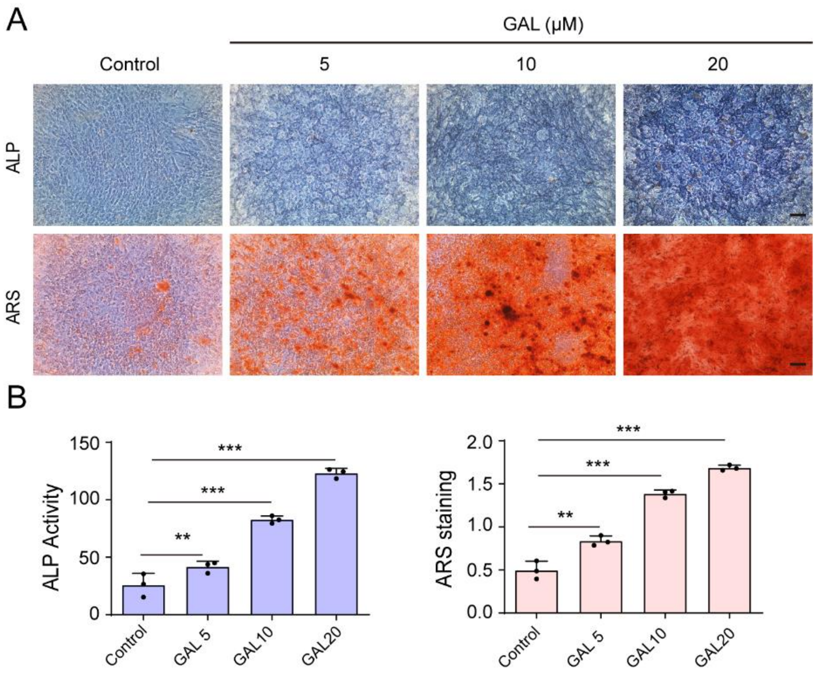

Supplementary Figure S3. GAL dose-dependently promotes osteogenic differentiation in human BMSCs (A,B) BMSCs were treated with different concentrations of GAL in osteogenic medium for 7 days and 14 days and then subjected to ALP activity assays and ARS staining. Scale bar: 100 μm. Data are shown as the mean ± SD (n = 3). $^{**}P < 0.01$, $^{***}P < 0.001$.
